# Organoids in ovarian cancer: a platform for disease modeling, precision medicine, and drug assessment

**DOI:** 10.1007/s00432-024-05654-0

**Published:** 2024-03-20

**Authors:** Haiyao Hu, Chong’en Sun, Jingyao Chen, Zhengyu Li

**Affiliations:** 1 Department of Obstetrics and Gynecology, West China Second University Hospital, Sichuan University, Chengdu, China; 2 Key Laboratory of Obstetrics and Gynecologic and Pediatric Diseases and Birth Defects of Ministry of Education, West China Second University Hospital, Sichuan University, Chengdu, China; 3 State Key Laboratory of Biotherapy and Cancer Center, West China Hospital, Sichuan University, Chengdu, China

**Keywords:** Ovarian cancer, Organoid, Cancer modeling, Precision medicine

## Abstract

Ovarian cancer (OC) is a major cause of gynecological cancer mortality, necessitating enhanced research. Organoids, cellular clusters grown in 3D model, have emerged as a disruptive paradigm, transcending the limitations inherent to conventional models by faithfully recapitulating key morphological, histological, and genetic attributes. This review undertakes a comprehensive exploration of the potential in organoids derived from murine, healthy population, and patient origins, encompassing a spectrum that spans foundational principles to pioneering applications. Organoids serve as preclinical models, allowing us to predict how patients will respond to treatments and guiding the development of personalized therapies. In the context of evaluating new drugs, organoids act as versatile platforms, enabling thorough testing of innovative combinations and novel agents. Remarkably, organoids mimic the dynamic nature of OC progression, from its initial formation to the spread to other parts of the body, shedding light on intricate details that hold significant importance. By functioning at an individualized level, organoids uncover the complex mechanisms behind drug resistance, revealing strategic opportunities for effective treatments.

## Introduction

Ovarian cancer (OC) is the most lethal malignant gynecological tumor and ranks eighth in both cancer incidence and mortality in women worldwide (Sung et al. 2021). Most patients with OC are diagnosed at advanced stages. More research effort on the molecular mechanism of ovarian tumorigenesis and tumor evolution is urgently needed. The standardized treatment management for OC consists of cytoreductive surgery, chemotherapy, and targeted therapy. Unfortunately, resistance to platinum-based chemotherapy, antiangiogenic targeted therapy, and poly-ADP-ribose polymerase inhibitors (PARPi)-targeted therapy is common, leading to 10-year survival of 15% in patients with advanced disease (Narod 2016; Colombo et al. 2019). The response of immune checkpoint inhibitor (ICI) monotherapy in patients with OC is unsatisfactory, and the combination of ICI and chemotherapy was also disappointing (Hamanishi et al. 2015; Matulonis et al. 2019; Kandalaft et al. 2022). Improved combined therapy to maximize medication is the goal of current research on OC. Prediction of response to platinum-based chemotherapy and PARPi is limited in certain genetic characteristics, such as patients with homologous recombination deficiency (HRD) benefit the most from PARPi treatment (Pujade-Lauraine et al. 2017; Swisher et al. 2017) and patients with CCNE1 amplification show intrinsic resistance to platinum-based chemotherapy (Patch et al. 2015; Walton et al. 2017). It is also unclear whether the maintenance-targeted therapy increases sensibility to platinum-based chemotherapy. This calls for real-time and accurate predictive models to guide individualized and precise treatment strategies.

In the realm of cancer research, various three-dimensional models have found increasingly widespread application within the field of cancer research. These 3D models exhibit superior performance compared to conventional research models in terms of design flexibility and cultivation stability (Loessner et al. 2010). The convergence of biomedical science and engineering has led to the continuous evolution and refinement of 3D models, encompassing spheroids, scaffold-free models, organoids, and 3D bioprinting. Spheroids are relatively straightforward to create. Nevertheless, they exhibit limitations in recapitulating the inherent heterogeneity of tumor cells and present challenges with regard to prolonged in vitro maintenance, limiting their utility in certain research contexts (Han et al. 2021; Zanoni et al. 2020). 3D bioprinting offers precise control over cell placement and facilitates the study of mechanical forces in tumor growth, but this technology remains in its early stages of development, primarily due to the prohibitive equipment costs associated with it (Yee et al. 2022). On the contrary, scaffold-free models, while easier to generate, lack crucial cell–extracellular matrix (ECM) interactions that play pivotal roles in the progression of cancer. This limitation can restrict their ability to faithfully mimic the intricacies of tumor behavior (Yee et al. 2022). In contrast, organoids represent a significant advancement in 3D models for cancer research. Organoids, as commonly understood in the context of this review, represent three-dimensional, self-renewing, and self-organizing structures derived from tumor cells sourced from patients or animal models, primarily following the widely adopted approach where these structures are embedded within a gel-like matrix that closely mimics the natural tumor microenvironment (Sato et al. 2009). It is worth noting that organoids possess the ability to closely mimic the intricacies and heterogeneity of primary tumors, retaining the genetic and molecular features of the tumor, such as mutations, gene expression, and drug resistance (Fan et al. 2019), which allows them to serve as invaluable tools for gaining insights into tumor behavior and evaluating potential therapeutic strategies.

**Fig. 1:**
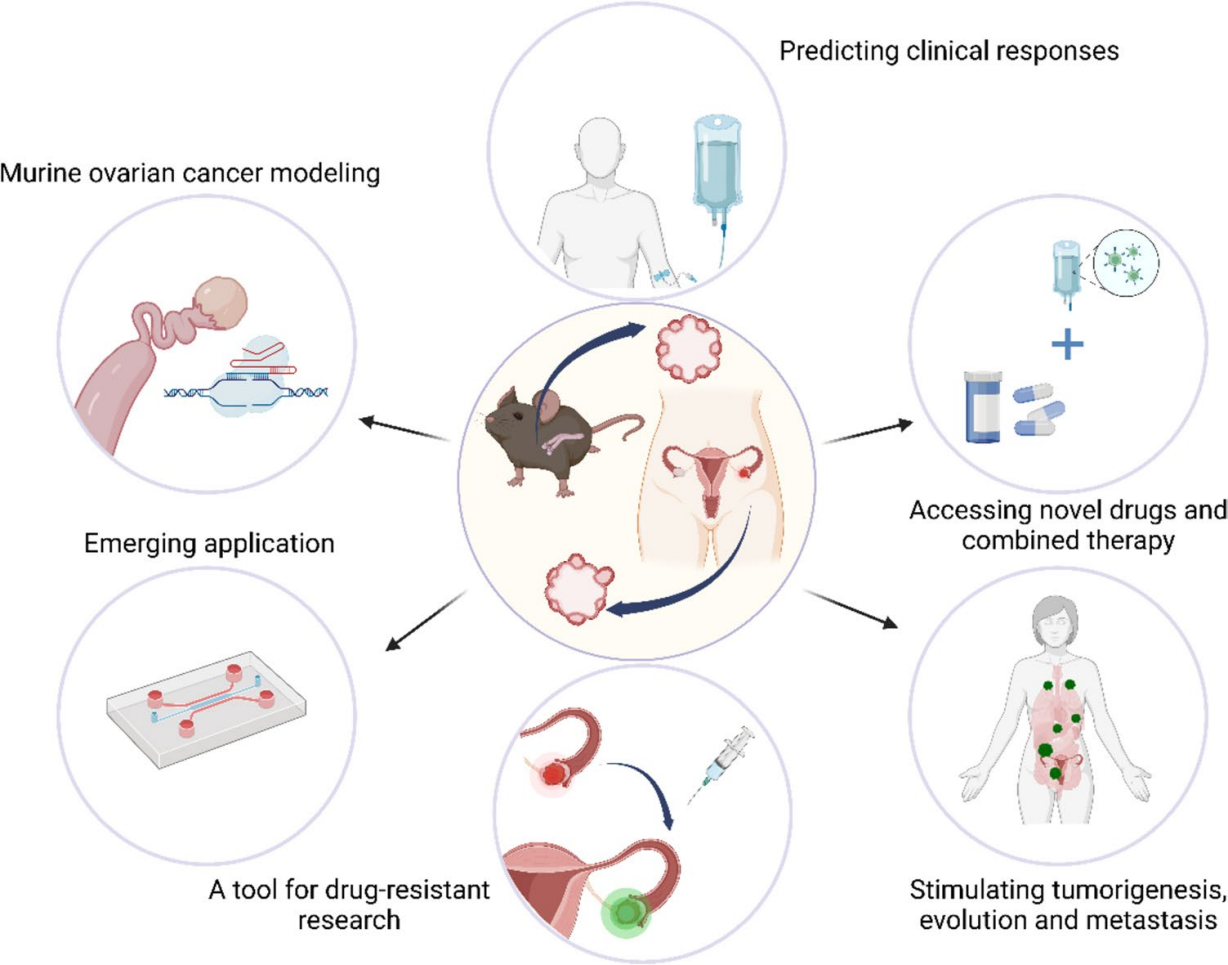
The application of murine Fallopian tube epithelium (FTE)/ovarian surface epithelium (OSE)-derived organoids and patient-derived organoids. Murine ovarian cancer modeling; predicting clinical responses; accessing novel drugs and combined therapy; stimulating tumorigenesis, evolution, and metastasis; a tool for drug-resistant research; emerging application

Combined with clustered regularly interspaced short palindromic repeats (CRISPR)/Cas9 technology, organoids serve as valuable tools for investigating tumor pathogenesis, drug screening, personalized treatment, and other related research (Matano et al. 2015; Drost et al. 2015; Hirt et al. 2022). In vitro cultured patient-derived organoids (PDOs) restore the pathological histochemical phenotype of tumors, and maintain gene stability and disease-state phenotype during long-term passage, offering the benefit of a shorter modeling time and higher fidelity to the primary tumor in comparison to conventional models (Dijkstra et al. 2018; Vlachogiannis et al. 2018). Long-term stable expansion is conducive to high-throughput drug screening, the development of new drugs for OC, and the exploration of combined therapy (Fig. 1).

While some scholars have previously conducted reviews on OC organoids, it is worth emphasizing that this field has experienced rapid development in recent years, giving rise to numerous novel and pertinent studies (Maru and Hippo 2019; Chang et al. 2022; Dumont et al. 2019; Yang et al. 2021). Here, our objective is to provide a clear and concise overview of the current status of organoid applications in OC, with a particular emphasis on innovative strategies for OC modeling, precision medicine, and drug assessment, presenting an up-to-date converge of the latest advancements in OC organoid research. More importantly, for the first time, we comprehensively summarize the ongoing clinical trials related to OC organoids.

## Murine fallopian tube epithelium (FTE)/ovarian surface epithelium (OSE)-derived organoids and human FTE organoids in cancer modeling

OC often onsets insidiously without obvious symptoms, making it difficult to diagnose at an early stage. Fundamental research on the tumor initiation of OC used to be limited by research models, such as gene-edited mouse models which are prone to non-intentional tumors and are time-consuming. Murine and human FTE/OSE organoids help to identify key molecular pathways and genetic alterations that drive tumor development and progression, which is more conducive to simulating realistic three-dimensional tumor growth than traditional 2D cell culture models and is less expensive and faster than xenograft models (Rae et al. 2021).

In an early study, normal mouse OSE organoids which were treated with hydrogen peroxide were transplanted into normal immune-intact mice, and early neoplastic changes were observed (King et al. 2013).

Recently, genetically engineered murine FTE-derived and OSE-derived organoids are capable of modeling different combinations of key driver mutations, presenting tumorigenesis tendencies in vitro. And, ovarian tumorigenesis was verified after organoid transplantation to immunocompetent and immune-deficient mice in several studies. Interestingly, two studies claimed that at least three genetic mutations were necessary for tumor initiation, while one study observed tumor formation with only two genetic alternations. Different genotype combinations resulted in different pathological types of OC generation, high-grade serous ovarian cancer (HGSOC)-like tumors, and sarcoma-like tumor (Maru et al. 2021). Lõhmussaar et al. and Zhang et al. transplanted different genetically modified murine FTE organoids and OSE organoids into nude mice in situ or mammary fat pat, establishing tumorigenesis models of OC with important and co-mutant driving genes, elucidating the dual-origin hypothesis with the help of comparable organoids models, consistent with the results of multi-omics research (Lõhmussaar et al. 2020; Zhang et al. 2019). However, both studies reported that the FTE organoids evolved into malignant tumors more readily in vivo. Also, murine FTE organoids contribute to the research of tumor microenvironment. Neel et al. developed a platform of multiple murine Fallopian tube epithelial organoids of different genotypes (Tp53^−/−^; Ccne1^OE^; Akt2^OE^; Kras^OE^, Tp53^−/−^; Brca1^−/−^; Myc^OE^, Tp53^−/−^; Pten^−/−^; Nf1^−/−^), demonstrating the significant similarity among corresponding organoids, organoid-transplanted mouse models, and patients with HGSOC in drug response, secretome, and tumor immune microenvironment (Zhang et al. 2021). In addition, they developed a highly effective combined therapy of gemcitabine, anti-CTLA4 antibodies, and anti-PD-L1 antibodies for Ccne1-overexpressing HGSOC. The ability to rapidly generated highly representative murine FTE organoids and rapidly develop effective combined immunotherapy using existing Food and Drug Administration (FDA)-approved drugs highlighted the extremely important role in the research of molecular mechanisms and exploration of combined therapy of OC. McGonical et al. established murine FTE organoids with BRCA mutations for research in chemoprevention. Germline BRCA-mutated carriers face a significantly elevated risk of OC tumorigenesis compared to the general population. There is an urgent need for chemopreventive treatments as a supplement to preventive salpingo-oophorectomy (Lynch et al. 2013; McGonigal et al. 2023; Ponti et al. 2023).

In a study published by Kessler et al. (2015), long-term organoids were successfully established from normal human FTE. These PDOs contained secretory and ciliated cells and responded to physiological treatments of estrogen and progesterone in vitro, indicating that FTE organoids mimic the in vivo physiological processes. Induced pluripotent stem cells (iPSC)-derived organoids facilitate the presentation of major molecular changes contributing to conversion from FTE to OC, providing a more accurate representation of patient-specific tumor development and progression and modeling the effects of genetic and environmental factors on tumor growth and metastasis (Tiriac et al. 2018; Huang et al. 2015; Kim et al. 2019). Yucer et al. (2021) obtained iPSCs from healthy individuals and young OC patients carrying the BRCA1 mutation (BRCA1mut) and differentiated them into FTE organoids. They found that the BRCA1mut-FTE organoids exhibited a series of cell abnormalities consistent with tumor transformation compared to the control FTE organoids without BRCA1 mutation. After a continuous 1-year in vitro culture, the BRCA1mut-FTE organoids exhibited a further increase in the number of epithelial cells and enhanced structural abnormalities and transformed from a single layer of the epithelium to a disordered, multi-layered epithelium filling up the luminal spaces. Moreover, the BRCA1mut-FTE organoids were able to sustain and show cancerous characteristics in nude mice, while the normal organoids were not, demonstrating that BRCA1mut organoids indeed replicate the process of tumor generation. Hoffmann et al. (2020) derived FTE organoids from healthy human FTE and edited p53, PTEN, and RB in the organoid system. They screened for stable knockdown (KD) organoid systems through antibiotic selection and fluorescent labeling and subsequently cultivated them in various media, including FTE organoid culture medium (FTM) and OC minimal medium (OCM), which supplemented the fundamental organoid culture medium with BMP2 and EGF. The results showed that KD organoids were unable to grow long term in FTM, but could be stably amplified in OCM, exhibiting higher stemness and proliferative abilities, lower levels of differentiation marker expression, and similar phenotypic and functional characteristics to HGSOC organoids when cultured in OCM. These results suggest that a mere oncogenic mutation may not be sufficient to drive the malignant transformation of normal FTE. Dai et al. (2024) generated human FTE organoids with TP53 and RAD51D knockdown to investigate the dominant regulatory mechanism of RAD51D in the early carcinogenesis of OC.

The successful establishment of both murine and human FTE organoids, combined with genetic engineering technology, has provided a powerful tool for simulating the transition from genetically defective normal FTE cells to OC tumorigenesis both in vitro and in vivo under immune-proficient conditions.

## Application of patient-derived OC organoids in precision medicine

The most prevalent sources are tumor tissue samples or ascites obtained during the first interval debulking surgery or resection after neoadjuvant chemotherapy treatment (NACT). These PDOs, being directly derived from OC patients, offer the advantage of representing patient specificity, and research utilizing such PDOs usually emphasizes exploring their application in personalized medicine.

### The current state of establishing the PDO platform of OC

Successful PDO construction of all pathological subtypes of OC, including endometrioid ovarian cancer, high-grade serous, HGSOC, low-grade serous ovarian cancer, serous adenocarcinoma, serous borderline tumor, mucinous adenocarcinoma, mucinous borderline tumor, and mucinous cystadenocarcinoma, has been reported. These specific subtypes demonstrated high fidelity to their source tissues at the morphological, genomic, and single-cell transcriptome levels and exhibited consistent responses to carboplatin/paclitaxel comparable to those observed in corresponding subtypes of patients in the real-world clinical settings. Here, we present a table summarizing the status of PDO platform establishment in OC research (Table 1).

**Table 1:** PDO platform establishment in ovarian cancer research

Author, year	Number of organoid lines	Sample source	Tumor clinical stage/no	Representative Validation
Hill et al. (2018)	33	22 HGSOC tissues		H&E staining; immunohistochemistry; WES
Kopper et al. (2019)	56	13 HGSOC tissues; 5 LGSOC tissues; 3 SBT tissues; 2 MA tissues; 5 MBT tissues; 1 OCCC tissue; 1 ENOC tissue	6 Stage I; 2 Stage II; 13 Stage III; 9 Stage IV	H&E staining; IHC; WGS; RNA sequencing; genetic manipulation; drug screening
Chen et al. (2020)	4	Ascites or pleural effusion from 5 HGSOC patients; Ascites from 1 HGS peritoneal cancer patient	4 Stage III; 2 Stage IV	RNA sequencing; quantitative RT-PCR analysis; Ki67 staining; histologic analysis; drug sensitivity testing
Maenhoudt et al. (2020)	13	22 HGSOC tissues; 2 LGSOC tissues; 1 MC tissue; 1 OCCC tissue; 1 malignant mixed mesonephric tumor tissue	2 Stage I; 1 Stage II; 11 Stage III; 13 Stage IV	H&E staining; immunohistochemical and immunofluorescent analysis; WES; low-coverage WGS; qRT-PCR; drug sensitivity testing
Bi et al. (2021)	43	14 HGSOC tissues; 5 HGSOC ascites; 4 LGSOC tissues; 3 OCCC tissues; 1 carcinosarcoma tissue; 1 malignant Brenner tumor ascites	5 Stage I; 3 Stage II; 9 Stage III; 9 Stage IV; 2 Recurrent	H&E staining; IHC; drug sensitivity testing
Senkowski et al. (2023)	17	10 HGSOC tissues	4 Stage III; 6 Stage IV	WGS; IHC; single-cell RNA sequencing
Vias et al. (2023)	18	10 HGSOC tissues; 43 HGSOC ascites; 15 HGSOC patient-derived xenografts	9 Stage III; 6 Stage IV	H&E staining; IHC; single-cell DNA sequencing; mutation analysis; shallow whole-genome sequencing; drug sensitivity testing;

*HGS* high-grade serous, *HGSOC* high-grade serous ovarian cancer, *LGSOC* low-grade serous ovarian cancer, *SBT* serous borderline tumor, *MA* mucinous adenocarcinoma, *MBT* mucinous borderline tumor, *MC* mucinous cystadenocarcinoma, *OCCC* ovarian clear cell carcinoma, *H&E* hematoxylin and eosin, *IHC* immunohistochemistry, *WES* whole-exome sequencing, *WGS* whole-genome sequencing

Currently, a standardized set of culture conditions for OC organoids is lacking, prompting a growing number of studies to actively investigate more efficient culture parameters. An analysis and comparison of the culture media employed were conducted (Table 2). Furthermore, the absence of uniform success criteria for establishing an organoid platform for OC poses significant challenges to the prospective clinical translation of organoids.

**Table 2:** Comparative analysis of culture media components and success rates in five referenced studies

	Kopper et al. (2019)	Hoffmann et al. (2020)	Maenhoudt et al. (2020)	Bi et al. (2021)	Senkowski et al. (2023)	
					M1	M2
Derivation efficiency	55%	~ 30%	44%	75%	53%	
Glutamax	1×	1×	1×	1×	1×	1×
Pen/strep	0.2% (Primocin)	100 U ml^−1^/100 mg ml^−1^	1×	2% (Primocin)	Primocin 1×	Primocin 1×
A83-01	0.5 μM	–	0.25 μM	5 μM	0.5 μM	0.5 μM
Nicotinamide	10 mM	1 mM	5 mM	5 mM	5 mM	5 mM
N2 supplement	–	1×	1×	10 μM	–	–
B27 supplement	1×	1×	1×	1:50	1×	1×
N-acetylcysteine	1.25 mM	–	1.25 mM	1.25 mM	1 mM	1 mM
17-β estradiol	100 nM	–	10 nM	100 nM	100 nM	100 nM
p38i (SB203580)	–	0.5 μM	1 μM	–	0.5 μM	0.5 μM
EGF	5 ng/mL	10 ng/mL	50 ng/mL	50 ng/mL	–	5 ng/mL
FGF10	10 ng/mL	100 ng/mL	–	100 ng/mL	10 ng/mL	10 ng/mL
FGF4	–	–	–	–	10 ng/mL	10 ng/mL
Noggin	1%	100 ng/mL*	100 ng/mL	100 ng/mL	–	–
R-spo1	10%	25%*	50 ng/mL	250 ng/mL	–	–
IGF1	–	–	20 ng/mL	–	–	–
HGF	25 mg/mL*	–	10 ng/mL	–	–	–
Heregulin-β-1	37.5 ng/mL	–	50 ng/mL	37.5 ng/mL	–	37.5 ng/mL
WNT3A	20%*	–	–	–	–	–
Forskolin	10 μM	–	–	10 μM	–	5 μM
Hydrocortisone	500 ng/mL	–	–	500 ng/mL	–	500 ng/mL
Y27632	5 μM	9 μM	10 μM	10 μM	–	–
HEPES	10 mM	10 mM	–	10 mM	10 mM	10 mM

*varied with patients

Based on human FTE organoid medium, Kopper et al. added hydrocortisone, forskolin, and heregulin-β-1, with or without WNT condition medium, creating two culture conditions. They cultured cells from the same source in those different conditions at the same time and selected the better growth medium after 2–3 passages. The source cells encompassed all pathological types of ovarian cancer, and the overall derivatization success rate was 65%, of which the derivatization success rate of HGSOC was 55% (Kopper et al. 2019). Utilizing a human FTE organoid medium as a foundation, Kopper et al. supplemented it with hydrocortisone, forskolin, and heregulin-β-1, either with or without WNT condition medium, resulting in the creation of two distinct culture conditions. Simultaneously, cells sourced from various pathological types of OC were cultured in these divergent conditions, and the optimal growth medium was selected following 2–3 passages. The collective success rate of derivation across all pathological types was 65%, with a specific success rate of 55% observed for HGSOC (Kopper et al. 2019). Building upon the human endometrial cancer organoid medium, Maenhoudt et al. (2020) conducted a comprehensive assessment of various components, adjusting their proportions to formulate an ovarian cancer organoid medium. The source cells encompassed the majority of pathological types of ovarian cancer, and the reported derivation efficiency reached 56%. Based on the human endometrial cancer organoid medium, Maenhoudt et al. (2020) comprehensively evaluated a variety of components and adjusted the ratio to generate an OC organoid medium. The source cells include most of the pathological types of OC and claimed derivation efficiency of 56%. Hoffmann et al. (2020) made modifications to the medium developed by Kopper, resulting in a derivatization success rate of approximately 30%. During extended culture periods, they observed inevitable growth arrest, highlighting the advantageous nature of a low-Wnt culture environment for the establishment and growth of PDOs. In the investigation led by Bi et al. (2021), they proficiently established 21 organoid cultures out of 28 OC tumor tissues or ascites samples, claiming a success rate of 75%. Adjustments to proportions were made based on the medium developed by Kopper. In a recent study by Senkowski et al. (2023), two culture media tailored for HGSOC were developed and compared with previous culture conditions (Kopper, Maenhoudt), employing a parallel culturing strategy akin to Kopper’s approach. The authors advocate for stricter criteria than those previously established for defining successful organoid culture, asserting a success rate of 53%. These criteria necessitate stable passage for more than 10 generations, absence of growth stagnation, and simultaneous assurance of genomic fidelity and other relevant aspects.

In most studies of OC PDOs, the sample size of established organoid cultures is limited, and the success rate of establishment as well as long-term stable passage is usually not explicitly mentioned in the literature, with a lack of in-depth exploration into the reasons for failure. Having attained the proof-of-concept stage, future research on the construction of the PDO platform for OC should improve the success rate of stable passage and verify its long-term fidelity in vitro, so as to accelerate the clinical transformation of organoid-assisted precision personalized therapy. Moreover, the individualized culture conditions not only underscore the considerable heterogeneity among individuals with OC, but also suggest that cells that have not successfully derived may require distinct, yet-to-be-developed media formulations. Anticipated future developments may involve further subdivision of individual culture conditions to accommodate the requirements of diverse cellular populations.

One of the advantages of organoid models is their potential for multiple passages (Clevers 2016; Sato and Clevers 2013). However, an increase in passaging also introduces new challenges, such as in vitro clonal selection and epigenetic changes leading to phenotypic alterations (Drost et al. 2016; Gao et al. 2014). Among the studies encompassed in our analysis, the passage number for PDOs varies from 0 to over 30 times. When conducting drug screening to predict or guide clinical responses, it is advisable to keep the number of passages and cultivation time as short as possible (Chen et al. 2020; Bi et al. 2021). This approach allows for a wider clinical treatment window and avoids accumulating genetic background alterations as proven by an experiment (Hill et al. 2018) conducting whole-exome sequencing analysis of OC PDOs within their first two passages. Conversely, when PDO is applied in mechanistic studies, such as gene-editing experiments, it becomes necessary to leverage the organoids’ ability for multiple passages to expand enough clones, facilitating long-term, high-throughput experiments. Senkowski et al. conducted single-cell sequencing on OC PDOs that underwent several months of passages, revealing that organoids subjected to prolonged ex vivo cultivation can still maintain a promising level of strong fidelity. In addition, recent research has proven a notably high success rate (85–100%) in the cryopreservation and revival of OC PDOs (Kopper et al. 2019; Senkowski et al. 2023). If immediate drug-screening experiments or analogous assays are unnecessary, cryopreservation emerges as a viable alternative. This is due to its minimal impact on organoid amplification efficiency, concurrently alleviating adverse effects associated with clonal selection resulting from multiple passages.

Current research suggests that failure to establish OC PDOs may be due to the administration of NACT before sample acquisition and an excessively prolonged time interval between sample acquisition and culture which reduces the viability and proliferation of the stem cells (Bi et al. 2021). Collectively, large-scale platforms for deriving OC PDOs are currently feasible using the existing methodology, with the successful derivation of PDOs from all major histological subtypes of OC. Moreover, OC PDOs have been validated to represent tumor tissue with high specificity across various scales, including genomic composition, protein expression, cell morphology, drug sensitivity assays, and physiological functions.

### Predicting patients’ clinical responses

PDOs have been validated as promising tools for novel drug development and preclinical high-throughput drug screening, allowing the prediction of patients’ clinical responses prior to treatment and providing a basis for personalized treatment (Jabs et al. 2017). In a groundbreaking research conducted by Vlachogiannis et al. (2018), PDOs from 71 patients diagnosed with metastatic gastrointestinal cancer exhibited an exceptional 93% specificity, 100% sensitivity, 88% positive predictive value, and 100% negative predictive value, underscoring organoids’ considerable clinical significance. In another study that established 212 lung cancer PDOs, an overall concordance of 83.33% was observed between organoid drug sensitivity and clinical response (Wang et al. 2023). These large-scale experiments in other cancers indicate that PDOs also harbor the potential to forecast clinical responses in OC as well. The potential of PDOs has also been observed in several studies of OC. Jabs et al. compared PDOs and 2D cell culture systems derived from tumors, ascites, and pleural effusions of nine patients with ovarian serous adenocarcinoma. They found that PDOs exhibited drug responses that were more strongly correlated with patient genotypes, suggesting that OC PDOs have a greater capacity to simulate clinical responses in the real world (Jabs et al. 2017). However, these studies did not delve into whether individual organoids from a specific patient accurately predict the corresponding patient’s clinical response.

Increasing preclinical trials have validated the feasibility and utility of utilizing tumor organoids for a personalized assessment of drug response at the individual patient level. de Witte et al. (2020) conducted a study to demonstrate the ability of PDOs to predict clinical responses in OC patients. They defined patient response to clinical drugs based on Chemotherapy Response Score, CA-125 levels, and Response Evaluation Criteria in Solid Tumors (RECIST) criteria and defined the sensitivity of PDOs to drug response based on cell viability after drug treatment. They compared the sensitivity of organoids to cisplatin and paclitaxel with patient clinical response and found a high degree of consistency between the two. It is worth noting that they also established multiple organoids from different sites of the same patient (primary tumor, ascites, or metastasis) and compared their genomic features and drug responses. Heterogeneity was observed among organoids derived from different sites in terms of genetics and drug responses. Gorski et al. (2021) cultured surgical specimens from six patients with HGSOC into organoids. They defined the drug sensitivity of the PDOs by comparing their EC_50_ values with clinically achievable *C*_max_ values and defined patient clinical responses by progression-free survival (PFS) determined by RECIST criteria. They identified one PDO with an EC_50_ value above the clinically achievable plasma *C*_max_, which corresponded to a patient with significantly lower PFS than that of the others. This represents the first study using PFS as an indicator to demonstrate the potential of PDOs to predict clinical responses in OC patients.

Chen et al. reported a pioneering case wherein clinical treatment was guided using ascites-derived organoid. A 59-year-old patient with recurrent HGSOC was enrolled, whose ascites were derived to organoids for drug susceptibility testing, with IC50 being the sensitivity standard. The final treatment plan was formulated based on the patient’s medication history and drug sensitivity results. Following treatment, a reduction in CA125 levels, diminished metastases, and decreased ascites were observed. This case exemplifies the pivotal role of organoids in precision medicine, showcasing their robust representativeness, in vitro high-speed amplification, drug-screening capabilities, and the rapid identification of suitable drugs for drug-resistant patients (Chen et al. 2023). Gray et al. (2023) documented a case involving a patient with platinum-resistant, advanced LGSOC. A drug sensitivity assay performed on an organoid culture derived from the patient’s tumor informed the treatment plan. Subsequent to medication, the patient’s condition rapidly stabilized. Importantly, it is noteworthy that the drug susceptibility results did not align with the genetic test results, emphasizing the potency of organoid drug sensitivity as a valuable supplement to treatment when genomics fail to provide precise information.

Research of PDOs assessing clinical treatment expanded from validation of relevance to case reports. For recurrent patients with chemo-resistance, PDOs’ tests may be able to supplement standardized treatment and find potentially effective drugs or combination treatment regimens when clinical treatment is in a dilemma.

There are still some issues to be addressed, including (1) the limited sample size; (2) the lack of unified criteria to define patient clinical response and drug efficacy for PDOs; (3) the potential impact of confounding factors such as age and tumor clinical stage has not been considered; (4) due to the in vitro culture conditions of organoids, immune-related drugs and antiangiogenetic drugs cannot be evaluated at present; and (5) studies have shown that the drug sensitivity of organoids is related to the composition of the medium. In future, to validate and promote the clinical application of PDOs in predicting patient response, large-scale randomized controlled trials are required as supporting evidence, while unified criteria should be established.

Given the limited data available in previously published research, we have summarized clinical studies registered over the past few years as a supplementary compilation, which aimed to evaluate the relevance of drug responses of OC PDOs and clinical responses to standard chemotherapy. The application of these organoids in clinical cohorts has the potential to significantly integrate the molecular biology characteristics and treatment responses of cancer patients. This, in turn, may establish an efficacious platform for personalized precision treatment of OC. At present, eight clinical trials pertaining to OC organoids have been duly registered (Table 3).

**Table 3:** Registered clinical studies of PDO in OC

Register ID	Status/location	Estimated enrollment	Indication	Objective	Study design	Registration date
NCT05537844	Recruiting/UK	250	Ovarian cancer	Ascites-derived organoid establishment	Observational, prospective	2022/9/13
NCT05290961	Recruiting/China	30	Ovarian cancer	Drug-screening platform	Observational	2022/3/22
NCT04768270	Recruiting/China	30	Ovarian cancer	PDOs in precision therapy	Observational	2021/2/24
NCT05175326	Recruiting/China	64	Ovarian cancer	Evaluation of PDOs’ predictive accuracy	Observational, cohort	2022/1/3
NCT04555473	Recruiting/Italy	48	Ovarian, Fallopian tube, or primary peritoneal cancer	Evaluation of PDOs’ predictive accuracy	Observational, cohort	2020/9/18
NCT04279509	Unknown (was recruiting)/Singapore	35	Solid tumor (ovarian cancer included)	Evaluation of PDOs’ predictive accuracy	Interventional	2020/2/21
ChiCTR1800017767	Not yet recruiting/China	120	Ovarian cancer	Precise in vitro dynamic therapy guidance	Cause/relative factors study	2018/8/13

Data are from: https://trialsearch.who.int/, https://clinicaltrials.gov/, and https://www.chictr.org.cn/index.aspx

*PDO* patient-derived organoid, *PFS* progression-free survival, *OS* overall survival, *ORR* objective response rate, *DCR* disease control rate, *FIGO* International Federation of Gynecology and Obstetrics, *cRNAs* circulating RNA, *RECIST* Response Evaluation Criteria in Solid Tumors, *PDS* primary debulking surgery, *NACT* neoadjuvant chemotherapy, *IDS* interval debulking surgery

### Assessing novel drugs and combined therapy

The application of PDOs for high-throughput screening of novel drugs presents an opportunity to evaluate promising chemotherapeutics in a patient-specific context while preserving the genetic heterogeneity of the original tumor (Rae et al. 2021; Kopper et al. 2019). This strategy helps identify drug candidates that are most likely to be effective for individual patients, thus reducing the need for animal testing, while simultaneously elevating the level of evidence and expediting the drug development process. Shigeta et al. generated three PDO lines from tumors and ascites of two OCCC patients and performed high-throughput drug screening of 42 oncology drugs that have been approved by FDA or are in the later stages of development using two ascites-derived organoids. However, the promotion of this screening strategy is debatable due to the small sample size, which cannot effectively prove the homogeneity of organoids derived from the same patient's ascites and tumor tissue (even among different sites of tumor tissue) (Shigeta et al. 2021).

PDOs have been widely used not only for high-throughput drug screening but also for validation of candidate drugs, novel biomarkers, or combination therapies that have already been proven effective in cell lines, as they provide highly individualized and faithful in vitro models, accelerating the clinical translation of new drug development and combination therapies while ensuring research quality (McDowell et al. 2021; Vernon et al. 2020; Wambecke et al. 2021; Scattolin et al. 2020; Granchi et al. 2021; Liu et al. 2022; Wang et al. 2022a; Wan et al. 2022; Xuan et al. 2022).

Currently, research is focused on using PDO experiments as an important validation part for new drugs and combination therapies, laying a solid foundation for the precise application of personalized treatment with new drugs and combination therapies. When standard treatments do not apply to individuals in future, PDOs, as patient-specific, time-saving, and high-fidelity models, would timely guide precise clinical treatment for individuals, serving as an important supplement to standard treatment.

These investigations often involve the development of a limited number of PDOs to serve as representatives of clinical samples for the assessment of novel drugs or combination treatments. This approach, however, tends to overlook the inherent high heterogeneity of OC. Furthermore, none of these studies have employed a more precise genomic characterization method to validate the robust fidelity of organoids, and there is notable variation in the formulations of the culture media utilized.

### PDOs as a tool for drug-resistant OC research

Resistance to chemotherapy frequently occurs in OC, leading to a lack of response or relapse (Patch et al. 2015; Prados-Carvajal et al. 2021). PDOs maintain the genome and transcriptome similarity with the patient’s tumor tissues, providing an opportunity to investigate drug resistance mechanisms and test new drug combinations (Clevers 2016; Drost et al. 2016; Sachs et al. 2018).

McCorkle et al. cultured seven PDO lines from tumor tissues of seven OC patients. RNA sequencing was then performed to analyze the differences in gene expression between the paclitaxel-sensitive and -resistant organoids, revealing a significant upregulation of the ABCB1 gene in the resistant organoids (McCorkle et al. 2021). Sun et al. established ten PDOs from four platinum-resistant and six platinum-sensitive OC patients’ tumor tissues and identified Aurora-A as a resistant target through RNA sequencing analysis (Sun et al. 2020). As previously mentioned, Gorski et al. (2021) established six PDOs using tumor tissues from HGSOC patients, of which one exhibited carboplatin resistance. By comparing the mutational analysis results of this resistant PDO with the other five sensitive PDOs, they discovered that TEME178B may be a previously unreported gene associated with chemotherapy resistance. Wang et al. established a PDO platform using fresh tumor tissues from HGSOC patients. The organoids were divided into two groups: one group was treated with increasing concentrations of cisplatin to establish cisplatin-resistant organoids, which imitated the process from sensitivity to resistance in patients over a short period of time in vitro, while the other group was treated with physiological saline. Then the researchers performed RNA sequencing to analyze the differential gene expression between cisplatin-sensitive and cisplatin-resistant organoids, identifying fibrillin-1 as a target that contributes to chemoresistance (Wang et al. 2022b).

**Fig. 2:**
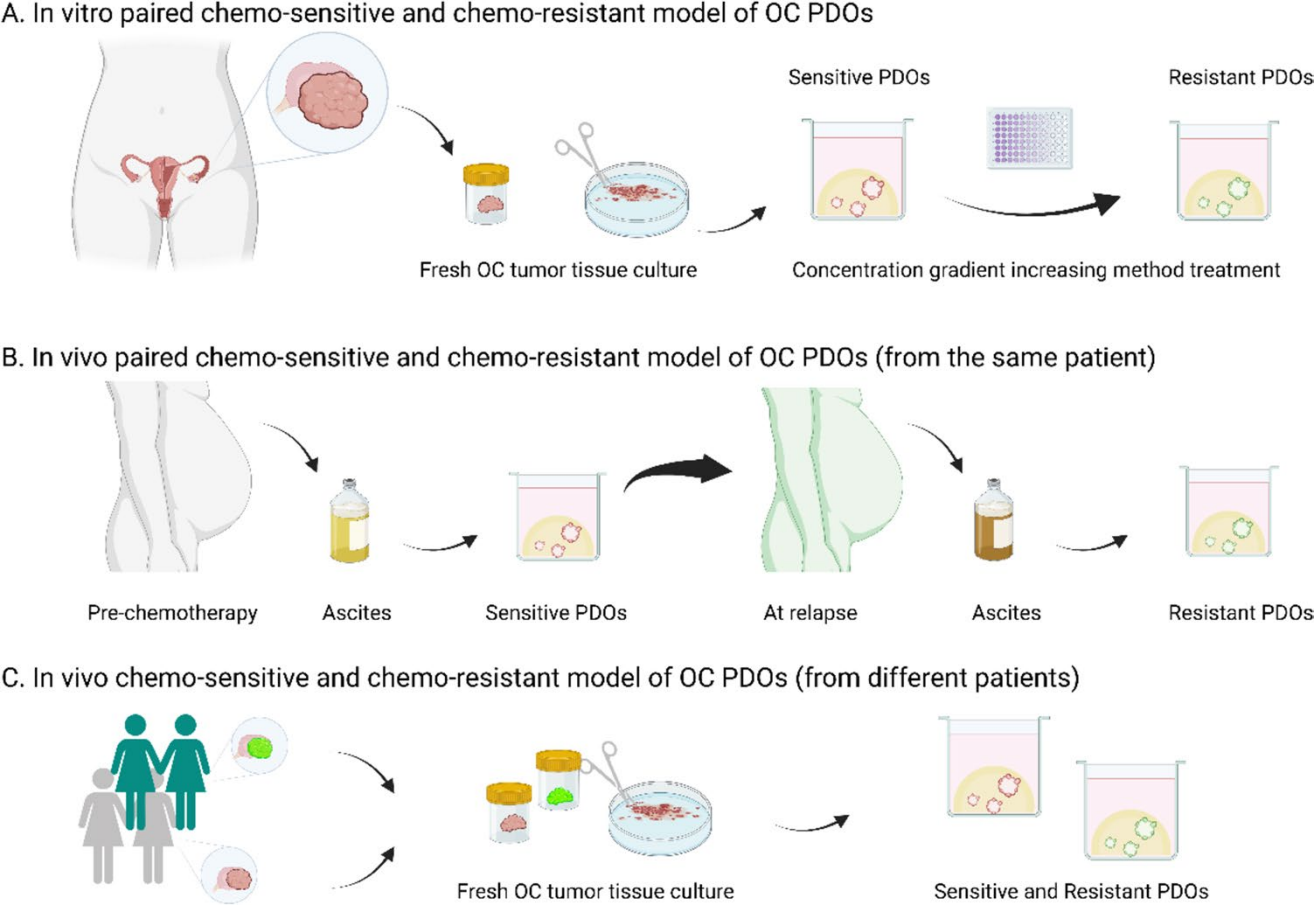
The methods of acquiring resistant PDOs. **A** In vitro paired chemo-sensitive and chemo-resistant model of OC PDOs; **B** In vivo paired chemo-sensitive and chemo-resistant model of OC PDOs (from the same patient); **C** In vivo chemo-sensitive and chemo-resistant model of OC PDOs (from different patients). *PDO* patient-derived organoid, *OC* ovarian cancer

PDOs have numerous advantages in the study of drug resistance mechanisms. They provide a controllable experimental platform that allows researchers to mimic the development process of drug resistance (Fig. 2). In the two distinct research modalities, the utilization of the concentration gradient increasing method to convert originally sensitive PDO into resistant PDO enables the acquisition of pairs of sensitive or resistant PDOs from the same sample, which facilitates the analysis of differences in their gene expression profiles. The results obtained from this approach minimize interference from other confounding factors, while also preserving the high representativeness of individual patients, thereby revealing the enormous potential of PDOs in modeling drug resistance. However, they lack consideration of the tumor microenvironment, including the effect of immune cells, stromal cells, and fibroblasts. Another constraint of this research paradigm is that despite the theoretical ability of organoids to be indefinitely passaged, the effect of passaging on organoids is intricate and may trigger alterations in their structure or phenotype. Therefore, to guarantee the stability and dependability of organoid culture and application, it is imperative to carefully regulate the number of passages (Hu et al. 2018; Yamamoto et al. 2017).

An alternative research methodology involves obtaining paired samples from the same patient before and after recurrence and conducting short-term organoid culture in vitro. Pietilä et al. utilized this approach by culturing organoid samples from ascites collected from one HGSOC patient before chemotherapy and after relapse and performed subsequent research on platinum drug resistance. The tumor that relapses in the patient’s body acquires evolutionary traits within the actual tumor microenvironment, and short-term in vitro culture without passage avoids the impact of clonal selection (Pietilä et al. 2021).

In future research on OC, it is essential to combine the strengths of different models to overcome their limitations. These models include (1) organoids from patients who have developed resistance in the real world, (2) PDOs derived from drug-sensitive patients and then treated to be drug resistant, and (3) drug-resistant immune-competent mouse models. Such models will allow for a more comprehensive analysis of the tumor microenvironment and enhance our understanding of the mechanisms underlying drug resistance. In turn, this helps in identifying new therapeutic targets and personalized treatment strategies for OC patients.

### Emerging applications and future perspective of PDOs in OC research

Currently, the PDO culture technology is not yet fully developed, and the following limitations hinder the prevalence of organoid technology in large-scale applications: (i) excessive growth of normal epithelial cells; (ii) absence of immune cells, vascular cells, and stromal cells (Jabs et al. 2017), resulting in the incapability to accurately replicate the tumor immune environment and angiogenesis, among other factors; and (iii) the relatively high cost of the currently used medium (Matrigel) and low consistency (Hughes et al. 2010) due to uncertainty of its origin.

In the realm of OC immunotherapy, ICI monotherapies exhibit limited efficacy, with only a minority of patients benefiting. One of the impediments is the inhibition of the immune microenvironment, rendering it difficult for newly elicited anti-tumor cells to enter the tumor stoma. Combination therapies (ICI with chemotherapy, PARPi, or antiangiogenic agents) have been observed to show better efficacy (Konstantinopoulos and Cannistra 2021); however, further exploration is still needed. A co-culture system of PDOs and immune cells mimics the cellular and molecular complexity of the tumor microenvironment, including the interaction between tumor cells and immune cells, and thus allows for a more accurate representation of the mechanisms of resistance to immunotherapy. Currently, researchers have made attempts to establish such co-culture systems. For instance, in lung cancer and colorectal cancer, a platform for the specific analysis of tumor immune response has been established by co-culturing patient-derived organoids with peripheral blood lymphocytes (Dijkstra et al. 2018; Yuki et al. 2020). Wan et al. conducted a study where they cultured tumor cells from 12 HGSOC patients into organoids and co-cultured them with immune cells in a 3D matrix to simulate the tumor microenvironment of HGSOC. They found that simultaneous blockade of both PD-1 and PD-L1 immune checkpoint proteins enhanced activation and proliferation of T cells, inhibited tumor cell growth, and identified three important ICI targets (Wan et al. 2021). Although not utilizing a co-culture system, Cao et al. also examined the potential of PDOs for investigating the efficacy of immunotherapy in OC. They cultured PDOs derived from the tumor tissues of 30 patients with HGSOC in a medium supplemented with anti-PD-1 antibodies and found that PDOs with lower levels of stromal tumor-infiltrating mast cells (TIMs) exhibited superior effector functions of CD8^+^ T cells in response to PD-1 blockade, indicating that PD-1 blockade could restore dysfunctional CD8^+^ T cells in patients with low stromal TIMs. Conversely, the accumulation of stromal TIMs may promote resistance to PD-1 blockade and serve as a new marker of immunoresistance (Cao et al. 2021).

Another approach is the “organoid-on-a-chip” tissue engineering technique that combines organoids with microfluidic chips. This novel technology overcomes some of the constraints of the application of organoids, by exerting control over the behavior of stem cells and the cellular microenvironment through further refinement of the design. While chip technology allows for artificial construction and precise control, organoids enable the self-assembly of tumor cells according to their innate developmental program. The convergence of these two distinct yet complementary technologies has garnered widespread acceptance among researchers. Although the cultivation mode for organoid-on-a-chip and well plates is similar, organoid-on-a-chip offers various advantages, such as integration, compact size, high throughput, ease of adaptation to detection devices, faster and more convenient sample addition, and improved stability of the cultivation system. Consequently, it offers a rapid and reliable platform for drug screening in precision cancer treatment (Cui et al. 2022; Hu et al. 2021). Although relatively underutilized in OC research, this technique has been investigated in other types of cancer, including liver, breast, and pancreatic cancers (Haque et al. 2021; Azimian Zavareh et al. 2022; Sun et al. 2019). In addition, the utilization of microfluidic technology may facilitate the incorporation of organoids and other stromal cells into microfluidic chips, thus enabling in vitro cultivation that more closely emulates the fluid circulation background of the in vivo setting (Zhu et al. 2023). In August 2022, the US FDA approved a novel indication for Sutimlimab, based solely on preclinical efficacy data obtained from organoid-on-a-chip research studies, along with pre-existing safety data, and allowed it to advance into clinical trials (NCT04658472). This approval indicates the acknowledgment of the reliability of organoid studies. One of the primary reasons for the high rate of failure for investigational therapies in the clinical development stage is the inability of animal models used in preclinical studies to accurately forecast human responses. The use of organoids can better emulate human responses to potential therapies, resulting in more innovations in the realm of biomedicine (Rumsey et al. 2022).

## Conclusion

Recently, organoid technology has emerged as a powerful tool in the field of oncology research. We summarized the application of murine-derived organoids, human FTE organoids, and PDOs in OC research. Murine FTE/OSE organoids help establish a syngeneic immune-intact primary OC model, recapitulating tumorigenesis and tumor evolution of different genotypes, also serving as an improved in vivo model for tumor immune microenvironment research to exploit immune combined therapy. Human FTE organoids and PDOs are a promising 3D tool for OC research since they mimic the tumor characteristics in terms of structure and histology and express specific gene phenotypes of patients. To date, PDOs have been verified as a promising model for novel drug development and preclinical high-throughput drug screening, though successful predictions of OC patients’ clinical responses have only been reported in a limited number of cases. Based on known mechanisms, PDOs can also be applied to testing the efficacy of new drugs, providing evidence for further clinical trials. With the advantage of simulating primary tumors, PDOs also serve as a tool for mechanism study in tumorigenesis, promotion, metastasis, and drug resistance of OC. The organoid platform pictures a promising landscape for cancer research, from delving into relevant mechanisms to application in personalized medicine, especially when combined with other advanced technologies.

## Data Availability

Not applicable.

## Abbreviations

OC: Ovarian cancer
PARPi: Poly-ADP-ribose polymerase inhibitor
ICI: Immune checkpoint inhibitor
HRD: Homologous recombination deficiency
ECM: Extracellular matrix
CRISPR: Clustered regularly interspaced short palindromic repeats
PDO: Patient-derived organoid
FTE: Fallopian tube epithelium
OSE: Ovarian surface epithelium
HGSOC: High-grade serous ovarian cancer
FDA: Food and Drug Administration
NACT: Neoadjuvant chemotherapy treatment
RECIST: Response Evaluation Criteria in Solid Tumors
PFS: Progression-free survival
iPSC: Induced pluripotent stem cell
KD: Knockdown
FTM: Fallopian tube organoid culture medium
OCM: Ovarian cancer minimal medium
TIMs: Tumor-infiltrating mast cells

## References

[CR1] AzimianZavareh V, Rafiee L, Sheikholeslam M, Shariati L, Vaseghi G, Savoji H et al (2022) Three-dimensional in vitro models: a promising tool to scale-up breast cancer research. ACS Biomater Sci Eng 8(11):4648–4672 36260561 10.1021/acsbiomaterials.2c00277

[CR2] Bi J, Newtson AM, Zhang Y, Devor EJ, Samuelson MI, Thiel KW et al (2021) Successful patient-derived organoid culture of gynecologic cancers for disease modeling and drug sensitivity testing. Cancers (Basel) 13(12):2901 34200645 10.3390/cancers13122901 PMC8229222

[CR3] Cao K, Zhang G, Zhang X, Yang M, Wang Y, He M et al (2021) Stromal infiltrating mast cells identify immunoevasive subtype high-grade serous ovarian cancer with poor prognosis and inferior immunotherapeutic response. Oncoimmunology 10(1):1969075 34527431 10.1080/2162402X.2021.1969075 PMC8437532

[CR4] Chang YH, Wu KC, Harnod T, Ding DC (2022) The organoid: a research model for ovarian cancer. Tzu Chi Med J 34(3):255–260 35912056 10.4103/tcmj.tcmj_63_21 PMC9333109

[CR5] Chen H, Gotimer K, De Souza C, Tepper CG, Karnezis AN, Leiserowitz GS et al (2020) Short-term organoid culture for drug sensitivity testing of high-grade serous carcinoma. Gynecol Oncol 157(3):783–792 32253045 10.1016/j.ygyno.2020.03.026 PMC7819712

[CR6] Chen W, Fang PH, Zheng B, Liang Y, Mao Y, Jiang X et al (2023) Effective treatment for recurrent ovarian cancer guided by drug sensitivity from ascites-derived organoid: a case report. Int J Womens Health 15:1047–1057 37465723 10.2147/IJWH.S405010 PMC10351532

[CR7] Clevers H (2016) Modeling development and disease with organoids. Cell 165(7):1586–1597 27315476 10.1016/j.cell.2016.05.082

[CR8] Colombo N, Sessa C, du Bois A, Ledermann J, McCluggage WG, McNeish I et al (2019) ESMO-ESGO consensus conference recommendations on ovarian cancer: pathology and molecular biology, early and advanced stages, borderline tumours and recurrent disease†. Ann Oncol 30(5):672–705 31046081 10.1093/annonc/mdz062

[CR9] Cui Y, Xiao R, Zhou Y, Liu J, Wang Y, Yang X et al (2022) Establishment of organoid models based on a nested array chip for fast and reproducible drug testing in colorectal cancer therapy. Bio-Des Manuf 5(4):674–686

[CR10] Dai Y, Xu J, Gong X, Wei J, Gao Y, Chai R et al (2024) Human fallopian tube-derived organoids with TP53 and RAD51D mutations recapitulate an early stage high-grade serous ovarian cancer phenotype in vitro. Int J Mol Sci 25(2):886 38255960 10.3390/ijms25020886 PMC10815309

[CR11] de Witte CJ, Espejo Valle-Inclan J, Hami N, Lõhmussaar K, Kopper O, Vreuls CPH et al (2020) Patient-derived ovarian cancer organoids mimic clinical response and exhibit heterogeneous inter- and intrapatient drug responses. Cell Rep 31(11):107762 32553164 10.1016/j.celrep.2020.107762

[CR12] Dijkstra KK, Cattaneo CM, Weeber F, Chalabi M, van de Haar J, Fanchi LF et al (2018) Generation of tumor-reactive T cells by co-culture of peripheral blood lymphocytes and tumor organoids. Cell 174(6):1586-1598.e12 30100188 10.1016/j.cell.2018.07.009 PMC6558289

[CR13] Drost J, van Jaarsveld RH, Ponsioen B, Zimberlin C, van Boxtel R, Buijs A et al (2015) Sequential cancer mutations in cultured human intestinal stem cells. Nature 521(7550):43–47 25924068 10.1038/nature14415

[CR14] Drost J, Karthaus WR, Gao D, Driehuis E, Sawyers CL, Chen Y et al (2016) Organoid culture systems for prostate epithelial and cancer tissue. Nat Protoc 11(2):347–358 26797458 10.1038/nprot.2016.006 PMC4793718

[CR15] Dumont S, Jan Z, Heremans R, Van Gorp T, Vergote I, Timmerman D (2019) Organoids of epithelial ovarian cancer as an emerging preclinical in vitro tool: a review. J Ovarian Res 12(1):105 31703723 10.1186/s13048-019-0577-2 PMC6839218

[CR16] Fan H, Demirci U, Chen P (2019) Emerging organoid models: leaping forward in cancer research. J Hematol Oncol 12(1):142 31884964 10.1186/s13045-019-0832-4 PMC6936115

[CR17] Gao D, Vela I, Sboner A, Iaquinta PJ, Karthaus WR, Gopalan A et al (2014) Organoid cultures derived from patients with advanced prostate cancer. Cell 159(1):176–187 25201530 10.1016/j.cell.2014.08.016 PMC4237931

[CR18] Gorski JW, Zhang Z, McCorkle JR, DeJohn JM, Wang C, Miller RW et al (2021) Utilizing patient-derived epithelial ovarian cancer tumor organoids to predict carboplatin resistance. Biomedicines 9(8):1021 34440225 10.3390/biomedicines9081021 PMC8394135

[CR19] Granchi C, Bononi G, Ferrisi R, Gori E, Mantini G, Glasmacher S et al (2021) Design, synthesis and biological evaluation of second-generation benzoylpiperidine derivatives as reversible monoacylglycerol lipase (MAGL) inhibitors. Eur J Med Chem 209:112857 33045662 10.1016/j.ejmech.2020.112857

[CR20] Gray HJ, Chatterjee P, Rosati R, Appleyard LR, Durenberger GJ, Diaz RL et al (2023) Extraordinary clinical response to ibrutinib in low-grade ovarian cancer guided by organoid drug testing. NPJ Precis Oncol 7(1):45 37202426 10.1038/s41698-023-00379-8 PMC10195827

[CR21] Hamanishi J, Mandai M, Ikeda T, Minami M, Kawaguchi A, Murayama T et al (2015) Safety and antitumor activity of anti-PD-1 antibody, nivolumab, in patients with platinum-resistant ovarian cancer. J Clin Oncol 33(34):4015–4022 26351349 10.1200/JCO.2015.62.3397

[CR22] Han SJ, Kwon S, Kim KS (2021) Challenges of applying multicellular tumor spheroids in preclinical phase. Cancer Cell Int 21(1):152 33663530 10.1186/s12935-021-01853-8 PMC7934264

[CR23] Haque MR, Rempert TH, Al-Hilal TA, Wang C, Bhushan A, Bishehsari F (2021) Organ-chip models: opportunities for precision medicine in pancreatic cancer. Cancers (Basel) 13(17):4487 34503294 10.3390/cancers13174487 PMC8430573

[CR24] Hill SJ, Decker B, Roberts EA, Horowitz NS, Muto MG, Worley MJ et al (2018) Prediction of DNA repair inhibitor response in short-term patient-derived ovarian cancer organoids. Cancer Discov 8(11):1404–1421 30213835 10.1158/2159-8290.CD-18-0474 PMC6365285

[CR25] Hirt CK, Booij TH, Grob L, Simmler P, Toussaint NC, Keller D et al (2022) Drug screening and genome editing in human pancreatic cancer organoids identifies drug-gene interactions and candidates for off-label treatment. Cell Genom 2(2):100095 35187519 10.1016/j.xgen.2022.100095 PMC7612395

[CR26] Hoffmann K, Berger H, Kulbe H, Thillainadarasan S, Mollenkopf HJ, Zemojtel T et al (2020) Stable expansion of high-grade serous ovarian cancer organoids requires a low-Wnt environment. EMBO J 39(6):e104013 32009247 10.15252/embj.2019104013 PMC7073464

[CR27] Hu H, Gehart H, Artegiani B, Löpez-Iglesias C, Dekkers F, Basak O et al (2018) Long-term expansion of functional mouse and human hepatocytes as 3D organoids. Cell 175(6):1591-1606.e19 30500538 10.1016/j.cell.2018.11.013

[CR28] Hu Y, Sui X, Song F, Li Y, Li K, Chen Z et al (2021) Lung cancer organoids analyzed on microwell arrays predict drug responses of patients within a week. Nat Commun 12(1):2581 33972544 10.1038/s41467-021-22676-1 PMC8110811

[CR29] Huang L, Holtzinger A, Jagan I, BeGora M, Lohse I, Ngai N et al (2015) Ductal pancreatic cancer modeling and drug screening using human pluripotent stem cell- and patient-derived tumor organoids. Nat Med 21(11):1364–1371 26501191 10.1038/nm.3973 PMC4753163

[CR30] Hughes CS, Postovit LM, Lajoie GA (2010) Matrigel: a complex protein mixture required for optimal growth of cell culture. Proteomics 10(9):1886–1890 20162561 10.1002/pmic.200900758

[CR31] Jabs J, Zickgraf FM, Park J, Wagner S, Jiang X, Jechow K et al (2017) Screening drug effects in patient-derived cancer cells links organoid responses to genome alterations. Mol Syst Biol 13(11):955 29180611 10.15252/msb.20177697 PMC5731348

[CR32] Kandalaft LE, DangajLaniti D, Coukos G (2022) Immunobiology of high-grade serous ovarian cancer: lessons for clinical translation. Nat Rev Cancer 22(11):640–656 36109621 10.1038/s41568-022-00503-z

[CR33] Kessler M, Hoffmann K, Brinkmann V, Thieck O, Jackisch S, Toelle B et al (2015) The Notch and Wnt pathways regulate stemness and differentiation in human fallopian tube organoids. Nat Commun 6:8989 26643275 10.1038/ncomms9989 PMC4686873

[CR34] Kim M, Mun H, Sung CO, Cho EJ, Jeon HJ, Chun SM et al (2019) Patient-derived lung cancer organoids as in vitro cancer models for therapeutic screening. Nat Commun 10(1):3991 31488816 10.1038/s41467-019-11867-6 PMC6728380

[CR35] King SM, Quartuccio SM, Vanderhyden BC, Burdette JE (2013) Early transformative changes in normal ovarian surface epithelium induced by oxidative stress require Akt upregulation, DNA damage and epithelial–stromal interaction. Carcinogenesis 34(5):1125–1133 23299406 10.1093/carcin/bgt003

[CR36] Konstantinopoulos PA, Cannistra SA (2021) Immune checkpoint inhibitors in ovarian cancer: can we bridge the gap between IMagynation and reality? J Clin Oncol 39(17):1833–1838 33891471 10.1200/JCO.21.00571

[CR37] Kopper O, de Witte CJ, Lõhmussaar K, Valle-Inclan JE, Hami N, Kester L et al (2019) An organoid platform for ovarian cancer captures intra- and interpatient heterogeneity. Nat Med 25(5):838–849 31011202 10.1038/s41591-019-0422-6

[CR38] Liu F, Tang L, Tao M, Cui C, He D, Li L et al (2022) Stichoposide C exerts anticancer effects on ovarian cancer by inducing autophagy via inhibiting AKT/mTOR pathway. Onco Targets Ther 15:87–101 35087279 10.2147/OTT.S340556 PMC8789324

[CR39] Loessner D, Stok KS, Lutolf MP, Hutmacher DW, Clements JA, Rizzi SC (2010) Bioengineered 3D platform to explore cell-ECM interactions and drug resistance of epithelial ovarian cancer cells. Biomaterials 32:8494–8506 10.1016/j.biomaterials.2010.07.064 20709389

[CR40] Lõhmussaar K, Kopper O, Korving J, Begthel H, Vreuls CPH, van Es JH et al (2020) Assessing the origin of high-grade serous ovarian cancer using CRISPR-modification of mouse organoids. Nat Commun 11(1):2660 32461556 10.1038/s41467-020-16432-0 PMC7253462

[CR41] Lynch HT, Snyder C, Casey MJ (2013) Hereditary ovarian and breast cancer: what have we learned? Ann Oncol 24(Suppl 8):viii83–viii95 24131978 10.1093/annonc/mdt313

[CR42] Maenhoudt N, Defraye C, Boretto M, Jan Z, Heremans R, Boeckx B et al (2020) Developing organoids from ovarian cancer as experimental and preclinical models. Stem Cell Rep 14(4):717–729 10.1016/j.stemcr.2020.03.004 PMC7160357 32243841

[CR43] Maru Y, Hippo Y (2019) Current status of patient-derived ovarian cancer models. Cells 8(5):505 31130643 10.3390/cells8050505 PMC6562658

[CR44] Maru Y, Tanaka N, Tatsumi Y, Nakamura Y, Yao R, Noda T et al (2021) Probing the tumorigenic potential of genetic interactions reconstituted in murine fallopian tube organoids. J Pathol 255(2):177–189 34184756 10.1002/path.5752

[CR45] Matano M, Date S, Shimokawa M, Takano A, Fujii M, Ohta Y et al (2015) Modeling colorectal cancer using CRISPR-Cas9-mediated engineering of human intestinal organoids. Nat Med 21(3):256–262 25706875 10.1038/nm.3802

[CR46] Matulonis UA, Shapira-Frommer R, Santin AD, Lisyanskaya AS, Pignata S, Vergote I et al (2019) Antitumor activity and safety of pembrolizumab in patients with advanced recurrent ovarian cancer: results from the phase II KEYNOTE-100 study. Ann Oncol 30(7):1080–1087 31046082 10.1093/annonc/mdz135

[CR47] McCorkle JR, Gorski JW, Liu J, Riggs MB, McDowell AB, Lin N et al (2021) Lapatinib and poziotinib overcome ABCB1-mediated paclitaxel resistance in ovarian cancer. PLoS One 16(8):e0254205 34347777 10.1371/journal.pone.0254205 PMC8336885

[CR48] McDowell A, Hill KS, McCorkle JR, Gorski J, Zhang Y, Salahudeen AA et al (2021) Preclinical evaluation of artesunate as an antineoplastic agent in ovarian cancer treatment. Diagnostics (Basel) 11(3):395 33652561 10.3390/diagnostics11030395 PMC7996621

[CR49] McGonigal S, Wu R, Grimley E, Turk EG, Zhai Y, Cho KR et al (2023) A putative role for ALDH inhibitors and chemoprevention of BRCA-mutation-driven tumors. Gynecol Oncol 176:139–146 37535994 10.1016/j.ygyno.2023.07.015 PMC10653209

[CR50] Narod S (2016) Can advanced-stage ovarian cancer be cured? Nat Rev Clin Oncol 13(4):255–261 26787282 10.1038/nrclinonc.2015.224

[CR51] Patch AM, Christie EL, Etemadmoghadam D, Garsed DW, George J, Fereday S et al (2015) Whole-genome characterization of chemoresistant ovarian cancer. Nature 521(7553):489–494 26017449 10.1038/nature14410

[CR52] Pietilä EA, Gonzalez-Molina J, Moyano-Galceran L, Jamalzadeh S, Zhang K, Lehtinen L et al (2021) Co-evolution of matrisome and adaptive adhesion dynamics drives ovarian cancer chemoresistance. Nat Commun 12(1):3904 34162871 10.1038/s41467-021-24009-8 PMC8222388

[CR53] Ponti G, De Angelis C, Ponti R, Pongetti L, Losi L, Sticchi A et al (2023) Hereditary breast and ovarian cancer: from genes to molecular targeted therapies. Crit Rev Clin Lab Sci 60(8):640–650 37455374 10.1080/10408363.2023.2234488

[CR54] Prados-Carvajal R, Irving E, Lukashchuk N, Forment JV (2021) Preventing and overcoming resistance to PARP inhibitors: a focus on the clinical landscape. Cancers (Basel) 14(1):44 35008208 10.3390/cancers14010044 PMC8750220

[CR55] Pujade-Lauraine E, Ledermann JA, Selle F, Gebski V, Penson RT, Oza AM et al (2017) Olaparib tablets as maintenance therapy in patients with platinum-sensitive, relapsed ovarian cancer and a BRCA1/2 mutation (SOLO2/ENGOT-Ov21): a double-blind, randomised, placebo-controlled, phase 3 trial. Lancet Oncol 18(9):1274–1284 28754483 10.1016/S1470-2045(17)30469-2

[CR56] Rae C, Amato F, Braconi C (2021) Patient-derived organoids as a model for cancer drug discovery. Int J Mol Sci 22(7):3483 33801782 10.3390/ijms22073483 PMC8038043

[CR57] Rumsey JW, Lorance C, Jackson M, Sasserath T, McAleer CW, Long CJ et al (2022) Classical complement pathway inhibition in a “human-on-a-chip” model of autoimmune demyelinating neuropathies. Adv Ther (Weinh) 5(6):2200030 36211621 10.1002/adtp.202200030 PMC9540753

[CR58] Sachs N, De Ligt J, Kopper O, Gogola E, Bounova G, Weeber F et al (2018) A living biobank of breast cancer organoids captures disease heterogeneity. Cell 172:373–386 29224780 10.1016/j.cell.2017.11.010

[CR59] Sato T, Clevers H (2013) Growing self-organizing mini-guts from a single intestinal stem cell: mechanism and applications. Science 340(6137):1190–1194 23744940 10.1126/science.1234852

[CR60] Sato T, Vries RG, Snippert HJ, van de Wetering M, Barker N, Stange DE et al (2009) Single Lgr5 stem cells build crypt-villus structures in vitro without a mesenchymal niche. Nature 459(7244):262–265 19329995 10.1038/nature07935

[CR61] Scattolin T, Bortolamiol E, Visentin F, Palazzolo S, Caligiuri I, Perin T et al (2020) Palladium(II)-η3-allyl complexes bearing *N*-trifluoromethyl *N*-heterocyclic carbenes: a new generation of anticancer agents that restrain the growth of high-grade serous ovarian cancer tumoroids. Chemistry 26(51):11868–11876 32368809 10.1002/chem.202002199

[CR62] Senkowski W, Gall-Mas L, Falco MM, Li Y, Lavikka K, Kriegbaum MC et al (2023) A platform for efficient establishment and drug-response profiling of high-grade serous ovarian cancer organoids. Dev Cell 58(12):1106-1121.e7 37148882 10.1016/j.devcel.2023.04.012 PMC10281085

[CR63] Shigeta S, Lui GYL, Shaw R, Moser R, Gurley KE, Durenberger G et al (2021) Targeting BET proteins BRD2 and BRD3 in combination with PI3K-AKT inhibition as a therapeutic strategy for ovarian clear cell carcinoma. Mol Cancer Ther 20(4):691–703 33509905 10.1158/1535-7163.MCT-20-0809 PMC8026742

[CR64] Sun W, Luo Z, Lee J, Kim HJ, Lee K, Tebon P et al (2019) Organ-on-a-chip for cancer and immune organs modeling. Adv Healthc Mater 8(4):e1801363 30605261 10.1002/adhm.201801363 PMC6424124

[CR65] Sun H, Wang H, Wang X, Aoki Y, Wang X, Yang Y et al (2020) Aurora-A/SOX8/FOXK1 signaling axis promotes chemoresistance via suppression of cell senescence and induction of glucose metabolism in ovarian cancer organoids and cells. Theranostics 10(15):6928 32550913 10.7150/thno.43811 PMC7295065

[CR66] Sung H, Ferlay J, Siegel RL, Laversanne M, Soerjomataram I, Jemal A et al (2021) Global cancer statistics 2020: GLOBOCAN estimates of incidence and mortality worldwide for 36 cancers in 185 countries. CA Cancer J Clin 71(3):209–249 33538338 10.3322/caac.21660

[CR67] Swisher EM, Lin KK, Oza AM, Scott CL, Giordano H, Sun J et al (2017) Rucaparib in relapsed, platinum-sensitive high-grade ovarian carcinoma (ARIEL2 Part 1): an international, multicentre, open-label, phase 2 trial. Lancet Oncol 18(1):75–87 27908594 10.1016/S1470-2045(16)30559-9

[CR68] Tiriac H, Belleau P, Engle DD, Plenker D, Deschênes A, Somerville TDD et al (2018) Organoid profiling identifies common responders to chemotherapy in pancreatic cancer. Cancer Discov 8(9):1112–1129 29853643 10.1158/2159-8290.CD-18-0349 PMC6125219

[CR69] Vernon M, Lambert B, Meryet-Figuière M, Brotin E, Weiswald LB, Paysant H et al (2020) Functional miRNA screening identifies wide-ranging antitumor properties of miR-3622b-5p and reveals a new therapeutic combination strategy in ovarian tumor organoids. Mol Cancer Ther 19(7):1506–1519 32371581 10.1158/1535-7163.MCT-19-0510

[CR70] Vias M, Morrill Gavarró L, Sauer CM, Sanders DA, Piskorz AM, Couturier DL et al (2023) High-grade serous ovarian carcinoma organoids as models of chromosomal instability. Elife 12:e83867 37166279 10.7554/eLife.83867 PMC10174694

[CR71] Vlachogiannis G, Hedayat S, Vatsiou A, Jamin Y, Fernández-Mateos J, Khan K et al (2018) Patient-derived organoids model treatment response of metastatic gastrointestinal cancers. Science 359(6378):920–926 29472484 10.1126/science.aao2774 PMC6112415

[CR73] Walton JB, Farquharson M, Mason S, Port J, Kruspig B, Dowson S et al (2017) CRISPR/Cas9-derived models of ovarian high grade serous carcinoma targeting Brca1, Pten and Nf1, and correlation with platinum sensitivity. Sci Rep 7(1):16827 29203787 10.1038/s41598-017-17119-1 PMC5715106

[CR74] Wambecke A, Ahmad M, Morice PM, Lambert B, Weiswald LB, Vernon M et al (2021) The lncRNA “UCA1” modulates the response to chemotherapy of ovarian cancer through direct binding to miR-27a-5p and control of UBE2N levels. Mol Oncol 15(12):3659–3678 34160887 10.1002/1878-0261.13045 PMC8637575

[CR75] Wan C, Keany MP, Dong H, Al-Alem LF, Pandya UM, Lazo S et al (2021) Enhanced efficacy of simultaneous PD-1 and PD-L1 immune checkpoint blockade in high-grade serous ovarian cancer. Cancer Res 81(1):158–173 33158814 10.1158/0008-5472.CAN-20-1674 PMC7878408

[CR76] Wan Y, Zhang Y, Meng H, Miao H, Jiang Y, Zhang L et al (2022) Bractoppin, a BRCA1 carboxy-terminal domain (BRCT) inhibitor, suppresses tumor progression in ovarian borderline tumor organoids. Biochem Biophys Res Commun 638:76–83 36442235 10.1016/j.bbrc.2022.11.063

[CR77] Wang W, Cho U, Yoo A, Jung CL, Kim B, Kim H et al (2022a) Wnt/β-catenin inhibition by CWP232291 as a novel therapeutic strategy in ovarian cancer. Front Oncol 12:852260 35646632 10.3389/fonc.2022.852260 PMC9134752

[CR78] Wang Z, Chen W, Zuo L, Xu M, Wu Y, Huang J et al (2022b) The Fibrillin-1/VEGFR2/STAT2 signaling axis promotes chemoresistance via modulating glycolysis and angiogenesis in ovarian cancer organoids and cells. Cancer Commun (Lond) 42(3):245–265 35234370 10.1002/cac2.12274 PMC8923131

[CR79] Wang HM, Zhang CY, Peng KC, Chen ZX, Su JW, Li YF et al (2023) Using patient-derived organoids to predict locally advanced or metastatic lung cancer tumor response: a real-world study. Cell Rep Med 4(2):100911 36657446 10.1016/j.xcrm.2022.100911 PMC9975107

[CR80] Xuan Y, Wang H, Yung MM, Chen F, Chan WS, Chan YS et al (2022) SCD1/FADS2 fatty acid desaturases equipoise lipid metabolic activity and redox-driven ferroptosis in ascites-derived ovarian cancer cells. Theranostics 12(7):3534–3552 35547771 10.7150/thno.70194 PMC9065188

[CR81] Yamamoto Y, Gotoh S, Korogi Y, Seki M, Konishi S, Ikeo S et al (2017) Long-term expansion of alveolar stem cells derived from human iPS cells in organoids. Nat Methods 14(11):1097–1106 28967890 10.1038/nmeth.4448

[CR82] Yang J, Huang S, Cheng S, Jin Y, Zhang N, Wang Y (2021) Application of ovarian cancer organoids in precision medicine: key challenges and current opportunities. Front Cell Dev Biol 9:701429 34409036 10.3389/fcell.2021.701429 PMC8366314

[CR83] Yee C, Dickson KA, Muntasir MN, Ma Y, Marsh DJ (2022) Three-dimensional modelling of ovarian cancer: from cell lines to organoids for discovery and personalized medicine. Front Bioeng Biotechnol 10:836984 35223797 10.3389/fbioe.2022.836984 PMC8866972

[CR84] Yucer N, Ahdoot R, Workman MJ, Laperle AH, Recouvreux MS, Kurowski K et al (2021) Human iPSC-derived fallopian tube organoids with BRCA1 mutation recapitulate early-stage carcinogenesis. Cell Rep 37(13):110146 34965417 10.1016/j.celrep.2021.110146 PMC9000920

[CR85] Yuki K, Cheng N, Nakano M, Kuo CJ (2020) Organoid models of tumor immunology. Trends Immunol 41(8):652–664 32654925 10.1016/j.it.2020.06.010 PMC7416500

[CR86] Zanoni M, Cortesi M, Zamagni A, Arienti C, Pignatta S, Tesei A (2020) Modeling neoplastic disease with spheroids and organoids. J Hematol Oncol 13(1):97 32677979 10.1186/s13045-020-00931-0 PMC7364537

[CR87] Zhang S, Dolgalev I, Zhang T, Ran H, Levine DA, Neel BG (2019) Both fallopian tube and ovarian surface epithelium are cells-of-origin for high-grade serous ovarian carcinoma. Nat Commun 10(1):5367 31772167 10.1038/s41467-019-13116-2 PMC6879755

[CR88] Zhang S, Iyer S, Ran H, Dolgalev I, Gu S, Wei W et al (2021) Genetically defined, syngeneic organoid platform for developing combination therapies for ovarian cancer. Cancer Discov 11(2):362–383 33158842 10.1158/2159-8290.CD-20-0455 PMC7858239

[CR89] Zhu J, Ji L, Chen Y, Li H, Huang M, Dai Z et al (2023) Organoids and organs-on-chips: insights into predicting the efficacy of systemic treatment in colorectal cancer. Cell Death Discov 9(1):72 36813783 10.1038/s41420-023-01354-9 PMC9947255

